# Amebic liver abscess in northern region of Bangladesh: sociodemographic determinants and clinical outcomes

**DOI:** 10.1186/1756-0500-7-625

**Published:** 2014-09-10

**Authors:** Faisal Alam, Md Abdus Salam, Pervez Hassan, Iftekhar Mahmood, Mamun Kabir, Rashidul Haque

**Affiliations:** Department of Microbiology, Rajshahi Medical College, Rajshahi, 6000 Bangladesh; Institute of Biological Sciences, University of Rajshahi, Rajshahi, 6205 Bangladesh; Department of Medicine, Kushtia Medical College, Kushtia, Bangladesh; Parasitology Laboratory, International Centre for Diarrhoeal Disease Research (ICDDR,B), Dhaka, Bangladesh

**Keywords:** Amebic liver abscess, *E. histolytica*, Sociodemographic determinants, Clinical outcomes, Bangladesh

## Abstract

**Background:**

Amebic liver abscess (ALA) is endemic in Bangladesh since historical age but its epidemiology and sociodemographic determinants are not well described in the literatures. This paper focuses on the endemicity, sociodemographic determinants and clinical outcomes of ALA patients from certain northern districts in Bangladesh. Ninety hospitalized ALA patients enrolled from 6 northern districts of Bangladesh during July 2008 to June 2010 were analyzed.

**Findings:**

Clinical presentations of ALA was initially substantiated by ultrasound imaging and later confirmed by detection of small subunit rRNA gene of *E. histolytica* using a Real Time PCR. Structured questionnaire and data sheet were used to record sociodemographic characteristics, clinical presentations and outcomes. Patients were followed for immediate and late treatment outcomes up to 2 years since diagnosis. Northern districts those situated on the Ganges basin were noted as endemic areas. Male significantly outnumbered the female with a male to female ratio of 21:1 and majority of patients (58%) were in their 3rd and 4th decades. A significant (21%) number of patients were aborigines despite their ethnic minority as population under investigation and overall 68% belonged to low socioeconomic group. Habit of indigenous alcohol consumption was very high (78%) among ALA patients with overwhelming majority was illiterate (74.44%) and from rural population (70%). Fever with right hypochondriac pain of variable duration was the principal presenting complains. Gross fluid derangements including pleural effusion, edema and ascities were observed in 39% cases and 6% had rupture of abscess. All patients were treated with standard antimicrobial regimen and discharged with initial recovery. Recurrent attack was observed in 6% cases and 3 (3.33%) patients died during 2 years follow-up period. Complicated (37.78%) ALA patients showed significant Odds ratio (P < 0.05) for major sociodemographic determinants in comparison to non-complicated patients.

**Conclusions:**

Amebic liver abscess is endemic in certain northern districts of Bangladesh especially on the Ganges basin with relatively high prevalence among aborigines. Rural habitat, ethnicity (Aborigine) and habit of indigenous alcohol consumption were found to be strong determinants, especially for complicated ALA, which were associated with different grades of morbidity and a few mortalities.

## Background

Amebic liver abscess (ALA) is an extra intestinal form of amebiasis and described since antiquity. It was first recognized as a deadly disease by Hippocrates (460–377 B.C.) and Losch first linked *E. histolytica* as a cause of this disease [1, 2]. In 1912, Leonard Rogers designated emetine as the first effective treatment for amebiasis [3]. Brompt revealed in 1925 that *E. histolytica* and *E. dispar* are morphologically identical but only *E. histolytica* is pathogenic for humans [4]. *E. histolytica* was first recognized as an agent of amebic liver abscess in 1945 by famous Robert Koch [5].

Global burden of amebiasis and amebic liver abscess has not been estimated precisely till to date. In older textbooks it is often stated that 10% of the world’s population is infected with *E. histolytica* but it is now known that at least 90% of these infections are due to *E. dispar* [4]. The last estimate on the global magnitude of this disease was made more than two decades ago [6]. According to WHO fact sheet, it is prevalent throughout the under developed and developing nations of the tropics with up to 50 million true *E. histolytica* infections and approximately 100,000 deaths occur each year mostly from liver abscesses or other complications [7]. Despite its medical importance, little is known about the current epidemiology of amebic liver abscess but it is assumed that the disease is prevalent within *E. histolytica* endemic countries. A few sporadic reports and hospital records claimed that amebic liver abscess is also endemic in Bangladesh but its exact annual incidence has not been figured out. International center for diarrhoeal diseases research, Bangladesh (ICDDR.B) has done a few extensive studies but those were mostly on intestinal amebiasis [8].

Rajshahi Medical College Hospital (RMCH) is a tertiary care teaching hospital homing for patients of many northern districts and its records show that a significant number of ALA patients are being treated over the years. A few investigators documented some sociodemographic aspects of these patients attended at RMCH, however, spectra of morbidity and mortality of ALA were not reported in those studies [9, 10]. More over, precise geographical distribution of ALA cases in the northern areas was not recorded by any investigator. This paper presents sociodemographic determinants, clinical presentations and treatment outcomes of amebic liver abscess patients with emphasis on its endemicity in certain northern districts of Bangladesh.

## Findings

### Methods

Clinically suspected liver abscess patients presented with fever and right hypochondriac pain those who were admitted into the Rajshahi Medical College Hospital, Bangladesh during July 2008 to June 2010 were included. Informed written consent was obtained from each patient and the protocol was approved by the Ethical Committee of the Institute of Biological Sciences (IBSc), University of Rajshahi, Bangladesh. All clinical and sociodemographic information of patients were recorded using structured questionnaires and data recording sheet. Privacy and confidentiality was maintained during collection, compilation and analysis of data.

Presence of space occupying lesion in the liver detected by ultrasound imaging substantiated initial clinical suspicion for ALA which was later confirmed as amebic liver abscess through detection of small-subunit rRNA (ssu rRNA) gene of *E. histolytica* in liver abscess aspirates by Real Time PCR. The number of confirmed ALA patients was 90.

### Real time PCR

Liver abscess pus (1 μL) was used as sample for PCR following standard procedure as described by Roy et al. [11]. The oligonucleotide primers and TaqMan probes (Eurogentec, United Kingdom) were designed to specifically amplify a 135 bp fragment inside the 16S-like small-subunit rRNA gene of *E. histolytica* (Gene Bank accession number X64142). Amplification reactions were performed in a volume of 25 μL with Qiagen super mix. Amplification results were analyzed using i-Cycler software.

## Results

### Epidemiology

**Figure 1:**
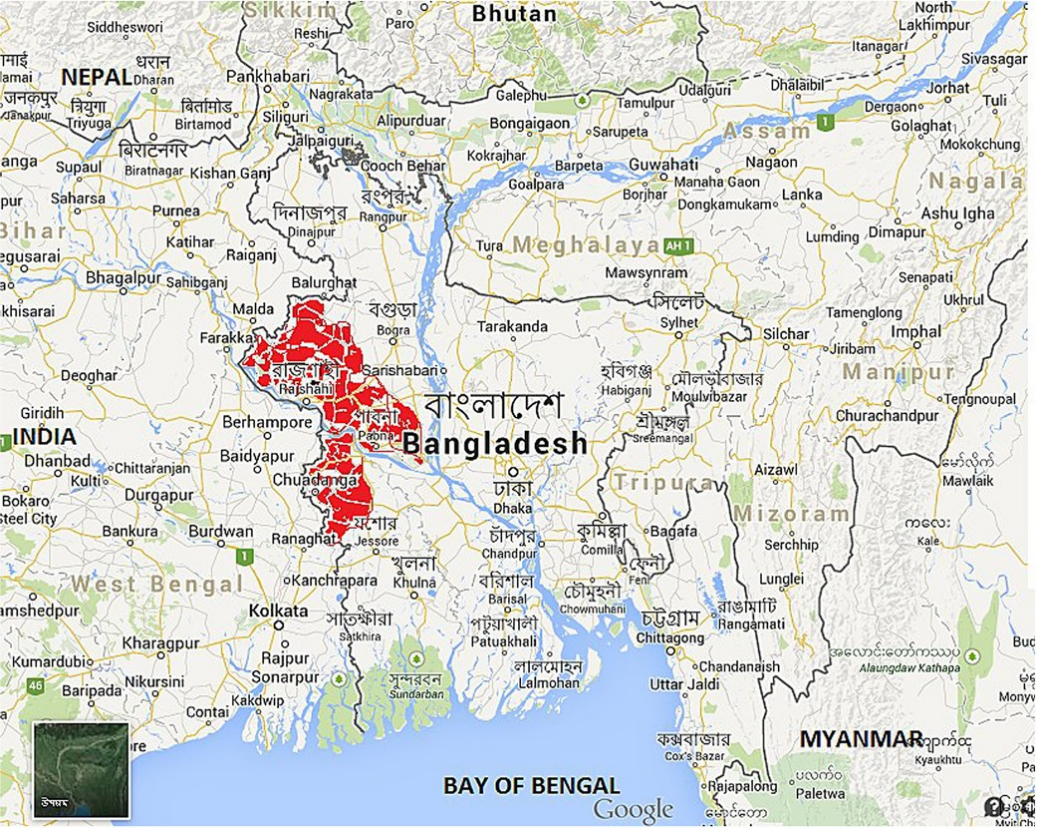
**Geographical distribution of amebic liver abscess endemic areas (marked in red color) of northern districts situated on the Ganges basin in Bangladesh.** (Source: https://www.google.com.bd/maps; accessed on 29/08/2014).

Amebic liver abscess patients of this study were from 6 northern districts situated in the Ganges basin of Bangladesh (Figure 1). The highest number of ALA cases was recorded from Rajshahi (34.44%) followed by Naogaon (27.78%), Chapainawabgonj (16.67%), Kushtia (10%), Natore (7.78%) and Pabna (3.33%).

### Sociodemographic characteristics

Gender distribution showed high male preponderance with a male to female ratio of approximately 21.5:1. Patients were grouped into 21–29 years, 30–39 years, 40–49 years, 50–59 years, and ≥ 60 years for their age groups with a mean age of 44.3 years. Majority of cases (58%) were from 3rd and 4th decade of their life. Overwhelming majority of ALA patients was illiterate (74.44%), from rural habitat (70%) with low socioeconomic (67.67%) condition and alcoholic (78.77%). A significant number of patients (21.11%) were from aborigine (Santal), which represents only about 2% of population under investigation. History of drug addiction (cannabis) was noted only in 2.22% patients (Table 1).

**Table 1:** **Sociodemographic profile of amebic liver abscess patients (n = 90)**

	n (%)
Gender	
*Male*	86 (95.56)
*Female*	4 (4.44)
*M:F ratio*	21.5:1
Mean age in years	44.3
Socioeconomic class	
*Low class*	60 (67.67)
*Lower middle class*	30 (32.33)
Education	
*Illiterate*	67 (74.44)
*Literate*	23 (25.56)
Habitat	
*Rural*	63 (70)
*Urban*	27 (30)
Ethnicity	
*Ethnic majority* (Inhabitant)	71 (78.89)
*Ethnic minority* (Aborigine)	19 (21.11)
Habit of Alcohol consumption	
*Alcoholics*	70 (77.78)
*Non-alcoholics*	20 (22.22)
Drug addiction	
*Addict*	02 (2.22)
*Non addict*	88 (97.78)

### Clinical presentations, complications and outcomes

Amebic liver abscess patients presented with fever (98.89%), right hypochondriac pain (76.67%), abdominal tenderness (97.78%) and dysentery (7.78%). Past history of dysentery and antibiotic intake before enrollment were noted in 13.33% and 70% cases respectively. A significant number of patients 29 (32.22%) developed hemodynamic changes (edema, effusion and ascites), while 4 (4.11%) persons had rupture of liver abscess and 1 (1.11%) had bronchopulmonary fistula. During 2 years post-treatment follow-up, 6 (6.67%) patients developed recurrent infections while 3 (3.33%) persons died (Table 2).

**Table 2:** **Clinical presentation, complications and treatment outcomes of amebic liver abscess patients (n = 90)**

	n (%)
Clinical features	
*Fever*	89 (98.89)
*Right hypochondriac pain*	69 (76.67)
*Abdominal tenderness*	88 (97.78)
*Dysentery*	07 (7.78)
Past history of	
*Dysentery*	12 (13.33)
*Antibiotics*	63 (70)
Complications	
*Hemodynamic changes* (edema, effusion & ascites)	29 (32.22)
*Rupture of liver abscess*	4 (4.44)
*Bronchopulmonary fistula*	1 (1.11)
Treatment outcomes	
*Initial recovery*	90 (100)
*Recurrent infections within 2 years follow-up*	6 (6.67)
*Death within 2 years follow-up*	3 (3.33)

### Correlation of sociodemographic determinants to the gravity of illness

ALA patients were divided into two groups; complicated and non-complicated in order to correlate the sociodemographic determinants to the gravity of illness. Out of 90 patients, 34 were categorized as complicated ALA cases who developed gross hemodynamic derangements, rupture of liver abscess and bronchopulmonary fistula. Complicated ALA cases showed significant odds ratio (P < 0.05) for major determinants like rural habitat, aborigine and alcohol consumption in comparison to 56 non-complicated ones (Table 3).

**Table 3:** **Correlation of sociodemographic determinants to the gravity of illness**

Determinants	Gravity of illness		Odds ratio	95% CI	P value
	Complicated ALA n (%)	Non-complicated ALA n (%)			
Gender					
Male	33 (97.06)	53 (94.64)	0.53	0.05–5.36	0.59
Female	01 (2.94)	03 (5.36)			
Ethnicity					
Aborigine	17 (50)	02 (3.57)	27.00	5.66–128.91	0.001
Native	17 (50)	54 (96.43)			
Habitat					
Rural	33 (97.06)	30 (53.57)	13.75	2.99–63.04	0.007
Urban	01 (2.94)	26 (46.43)			
Alcohol					
Alcoholic	29 (85.29)	41 (73.21)	16.95	2.15–133.62	0.007
Non alcoholic	05 (14.71)	15 (26.79)			

## Discussion

Historically amebiasis, both amebic colitis and amebic liver abscess have been endemic disease among people living in the Ganges basin of both India and Bangladesh probably due to the fact of fecal-oral transmission of these diseases. Catchment areas of the present study is within the Bangladeshi part of Ganges basin and our findings have corroborated with previous investigations and supports that ALA is endemic in certain northern districts of Bangladesh especially those situated in the Ganges basin [9, 10, 12].

Gender distribution in ALA cases shows a very high male preponderance and has coincided with most of the previous studies [13–15]. The fact that the gender discrimination can be correlated with iron deficiency state commonly present among rural females that brings an inhibitory effect to *E. histolytica* growth [16]. Further, complement mediated killing of *E. histolytica* was found more pronounced in female than male by some investigators [17] and failure to produce early cytokine like IFN γ was documented in case of male in mouse model [18]. Illiteracy, low socioeconomic condition and rural habitat are all coined together to poor hygiene and concomitant increased vulnerability to develop such pathology, which was also noted in other studies [19–21].

Most striking finding in the present investigation was a large number of cases (21%) were from small aborigine group which represents only 2.34% of total population in this study [22]. Few earlier local studies also showed such cluster of ALA cases among aborigines [9, 10]. Low socioeconomic condition with malnutrition, regular consumption of indigenous alcohol, which is a part of their social custom, poor sanitation as well as less access to health care facility are all essential factors that can be correlated with high ALA incidence among this ethnic minority population. Alcoholic persons develop fatty liver and low cellular immunity which lead to deposition of large amount of iron that facilitates *E. histolytica* growth in the liver parenchyma leading to liver abscess has been revealed by Makkar et al. [23] and this correlation has been reinforced in our findings support this too.

As far as the clinical presentations of ALA patients are concerned, overwhelming majority of patients presented with typical features of fever, right hypochondriac pain and abdominal tenderness of varying duration that are similar to other previous findings [10, 12, 14]. Association of concomitant dysentery (7.78%) or past history of dysentery (13.33%) were both noted in very low number of patients and similar low prevalence of colitis among ALA was also found by others [13, 15]. This raises the long controversial issue of whether same or different strains of parasite causing amebic colitis and liver abscess.

Different grades of morbidity including gross hemodynamic derangements (38.89%), rupture of abscess (4.44%) and bronchopulmonary fistula (1.11%) were noted among ALA patients and similar complications were also observed in other studies [12, 20]. Patients with these complications were categorized as complicated ALA and found to have strong correlation with major sociodemographic determinants like rural habitat, aborigine and alcohol consumption. All patients had initial cure with treatment by standard antimicrobial drugs but in the course of 2 years follow-up, 3 ALA cases died and 6 (6.67%) developed recurrent infections, which refer to fatal outcome as well as incomplete immunity involved in this chronic illness.

## Conclusions

Present investigation has documented that amebic liver abscess is prevalent and endemic in the northern districts situated on the Ganges basin in Bangladesh. Illiteracy, rural habitat, low socioeconomic condition, habit of indigenous alcohol consumption and male in their active age are important sociodemographic determinants for amebic liver abscess and majority of these factors has significant association with morbid complications in ALA. It leads to a significant number of morbidities and a few mortality, so early diagnosis and timely intervention are essential measures to reduce the detrimental outcomes. It is expected that findings of present investigation would help to bridge the knowledge gap in regards to the epidemiology, risk factors and clinical outcomes of amebic liver abscess.
